# Impact of a Combined Philosophy and Mindfulness Intervention on Positive and Negative Indicators of Mental Health Among Pre-kindergarten Children: Results From a Pilot and Feasibility Study

**DOI:** 10.3389/fpsyt.2020.510320

**Published:** 2020-12-11

**Authors:** Catherine Malboeuf-Hurtubise, David Lefrançois, Geneviève A. Mageau, Geneviève Taylor, Marc-André Éthier, Mathieu Gagnon, Carina DiTomaso

**Affiliations:** ^1^Department of Psychology, Faculty of Arts and Science, Bishop's University, Sherbrooke, QC, Canada; ^2^Department of Education, University of Quebec in Outaouais, Gatineau, QC, Canada; ^3^Department of Psychology, Université de Montréal, Montreal, QC, Canada; ^4^Department of Education and Pedagogy, Université du Québec à Montréal, Montreal, QC, Canada; ^5^Department of Didactics, Université de Montréal, Montreal, QC, Canada; ^6^Department of Preschool and Primary School Education, Université de Sherbrooke, Sherbrooke, QC, Canada

**Keywords:** preschool, school psychology, mental health, mindfulness-based interventions (MBIs), philosophy for children (P4C)

## Abstract

**Background:** Fostering greater resiliency to stress, optimal psychosocial development and promoting better mental health and well-being in youth is an important goal of the Canadian and American elementary school systems (1, 2). Recent research on mindfulness and philosophy for children (P4C) has yielded promising results regarding innovative interventions that may be implemented in elementary school settings to foster greater child resiliency and well-being (3–5).

**Goal:** The goal of this feasibility study was to pilot a new intervention, which combines mindfulness meditation and P4C activities, with the goal of improving mental health in pre-kindergarten children, assessed with positive (i.e., social skills and adaptability) and negative (i.e., internalized symptoms, comprises depression, anxiety, inattention; and hyperactivity) indicators.

**Methods:** A randomized cluster trial with a wait-list control group was employed to evaluate the impact of the combined MBI and P4C intervention on child mental health. Two classrooms of pre-kindergarten children (*N* = 38, mean age = 4.6 years old) took part in this study and were randomly allocated to the experimental or wait-list control conditions. Teachers completed pre- and post-intervention questionnaires.

**Results:** ANCOVAs did not reveal a significant effect of condition on internalized symptoms, controlling for baseline levels. Sensitivity analyses indicated that for the whole sample, internalized symptom scores were statistically significantly lower at post-intervention, when compared to pre-intervention scores. No impact of group on levels of hyperactivity was found, however, sensitivity analyses indicated that for both the experimental and control groups, hyperactivity scores were statistically significantly lower at post-intervention, when compared to pre-intervention scores. Finally, no impact of group on levels of social skills and adaptability were found. Sensitivity analyses conducted using paired t-tests did not indicate statistically significant pre-to-post changes in scores for both variables.

**Conclusion:** These preliminary results suggest that mindfulness and philosophy for children may not be the most effective intervention to foster short-term resiliency, well-being and better mental health in children. Yet, group differences were often small and past research suggested the effectiveness of this type of intervention. Further research considering the impact of moderators such as age or baseline levels of psychopathology, using longer time frames and comparing the effectiveness of this combined intervention with other types of school-based interventions with similar aims (such as, e.g., P4C or MBI alone) is warranted, to evaluate if mindfulness and P4C interventions have an added value compared to other types of interventions implemented in school settings.

## Introduction

Fostering greater resiliency to stress, optimal psychosocial development and promoting better mental health and well-being in youth is an important goal of the Quebec (Canadian province) and American elementary school systems (1, 2). In Canada, 20% of elementary school children have received a diagnosis of a psychological disorder (6). However, only a fifth of youth who need help actually have access to mental health services (7). Given the high prevalence of psychological difficulties in elementary school children, it appears crucial to develop interventions that can help them foster greater resiliency, better mental health and well-being. Resiliency is comprised of two essential components, which are (1) the presence of a significant risk/adversity (e.g., for one's own mental health); and (2) a positive adaptation in spite of this risk/adversity (8). Resiliency is considered to be a dynamic process, which results from interactions between personal attributes (e.g., emotional regulation skills, well-being, social skills, perseverance) and environmental risk and protective factors (8, 9).

In order to fully understand the concept of mental health, researchers have recently suggested to conceptualize it as the result of both positive (e.g., well-being and positive affect) and negative indicators (e.g., symptoms pertaining to psychopathology and negative affect) (10). Developing prevention strategies and interventions has been recommended as a potential solution to reach a greater number of students and to prevent psychological disorders, while fostering greater well-being (11). Recent research on mindfulness and philosophy for children has put forth promising results regarding innovative school interventions that may foster greater resiliency and well-being in elementary school children (3–5).

## Mindfulness-Based Interventions for Youth

In recent years, mindfulness-based interventions (MBIs) specifically designed for youth have received significant research attention (5, 12–14). Mindfulness can be defined as the process by which we bring our attention to the experience unfolding in the present moment, intentionally and without judgement (15). Results have shown that MBIs can significantly alleviate psychological distress and foster greater well-being and resiliency, namely in school settings (16–20). As such, MBIs could represent an interesting intervention to implement in school settings, namely because of their ease of implementation (21). MBIs typically consist of short exercises that are easily integrated within the classroom routine. These exercises are meant for students to develop their ability to pay attention and become aware of their sensory experiences and to recognize their thoughts and emotions, in order to become better at regulating them. Past research on MBIs in school settings has shown encouraging results with regards to decreasing various internalized (e.g., anxiety, depression, inattention) and externalized (e.g., aggressiveness, conduct disorders, hyperactivity) symptoms, while increasing well-being and resiliency both in regular education classrooms and special education classrooms (5, 12–14, 16–20, 22).

## Self-Determination Theory and Mindfulness

To date, there is a paucity of theoretical models explaining why and how MBIs have a positive impact on youth's well-being (21). In recent years, researchers have suggested that mindfulness and its effects on well-being could be best understood through self-determination theory [SDT; (23)]. SDT is a macro-theory of well-being and functioning that posits that we all have three basic psychological needs that are essential to well-being: the need for autonomy (the need to act with volition and in accordance with our interests and values), the need for competence (the need to have an impact on our environment) and the need for affiliation (feeling connected to others and loved by others). When measuring children's well-being, it is important to take into account the evaluations of relevant stakeholders in children's lives, but also children's own evaluations, and aspirations regarding their own lives. Basic psychological need satisfaction is a widely researched determinant of well-being among children. More than three decades of research with children show that the satisfaction of the basic psychological needs of autonomy, competence and relatedness in children is key for children's academic achievement, perseverance and life satisfaction, all of which have been included in various conceptualizations of child well-being (24). Hence, as SDT research will detail, during this early developmental period supports for relatedness, autonomy, and competence are required for infants and young children to be intrinsically motivated, to attach to others and form secure social bonds, and to integrate social regulations into their self-regulatory capacities, all processes essential to adaptation and thriving in “cultural animals” such as humans (25). Yet their importance goes beyond these early developmental issues, to bear on wellness, relationship qualities, experience, and quality of behavior in virtually every domain and at all ages across the lifespan.

The need for autonomy (also referred to as self-determination) is a cornerstone of SDT, and, as such, research has placed significant importance on the need for youth and adults alike to act volitionally and in accordance with their values. Research with adults has shown that mindfulness is positively correlated with self-determined motivation, which can be defined as doing an activity for the sake and pleasure of it or because it's personally meaningful and related to one's core values. Recently, mindfulness has been proposed as an antecedent of self-determined motivation (26), and evidence from correlational and priming studies shows that people who are more mindful report more self-determination in their day-to-day activities and choose to act in more self-determined ways (27–29). Several studies have even identified self-determination as a key explanatory mechanism in the relationship between mindfulness and well-being (29–32). Moreover, (33) showed that dispositional mindfulness increased well-being through the mediating role of self-determined goal motivation. Furthermore, initial research on students indicates that dispositional mindfulness was positively associated with self-determined motivation (34). However, these studies were mainly correlational and only focused on dispositional mindfulness as a predictor.

Recently, results from pilot studies using experimental designs have shown encouraging results as to how MBIs could be useful tools to foster self-determination in youth (35). Specifically, MBIs could help youth better identify and accept their personal values, strengths and weaknesses, thus leading to the construction of a more realistic identity and higher levels of self-determination and well-being (36). Furthermore, decades of research in SDT have indicated that higher self-determination is linked to perseverance and academic achievements in elementary and high school students (37, 38). However, results from (35, 39) study cited above, although encouraging, also indicated that the MBI was not more useful than a social skills development program to increase self-determination, which suggests that a MBI alone may be necessary, but not sufficient to significantly and undoubtfully improve basic psychological needs satisfaction in children. As such, we posit that the combination of mindfulness techniques with existential psychology (through philosophy for children activities, detailed below) could have a stronger impact on basic psychological needs satisfaction in elementary school students, particularly self-determination. Further explanation is provided below.

## Existential Psychology

Issues pertaining to self-determination, such as living a life in accordance with one's beliefs and values, are also central to existential psychology (40, 41). Existential psychology is a branch of clinical psychology in which individuals are encouraged to explore and discuss core existential questions and struggles that are shared by all humans. One goal of existential therapy is to bring the client to re-focus on the present and to notice what is important to them in the present. This renewed awareness is thought to help individuals become more self-determined and motivated to act in accordance with their values (42). Although existential psychology is different from SDT, within both theoretical frameworks, an individual's self-determination is central and can be achieved through increased awareness of one's own strengths, weaknesses, and other factors that can help or hinder self-determination, all of which can be facilitated through MBIs (43). Indeed, according to (43), SDT and existential psychology (as well as existential philosophers) share common grounds with regards to ideas related to authentic and inauthentic actions. Authentic actions can hence be seen as “endorsed by the self” and congruent with one's values and sense of self. Furthermore, Deci and Ryan (43) establish links between Kierkegaard's view of the self and SDT: “…to achieve a self is to be committed to relate the self to the self, of taking responsibility for ever reevaluating what one believes, and then acting in accord with that best synthesis. In this view, a genuine human being is “infinitely interested in his existence,” and what one does is the current best synthesis of all that one truly believes, knows, and feels.”

However, unlike research on SDT and mindfulness, evidence-based research within existential psychology for youth is almost inexistent (44). To our knowledge, no study to date has been published detailing the association and possible impact of an existential psychology intervention for children on well-being. Thus, one goal of this study was, to develop and present an adaptation of an intervention based on existential psychology for children, while integrating it with the SDT and mindfulness research frameworks in school settings. This was done in order to provide children with an intervention that is better suited to foster their self-determination. Indeed, as MBIs help individuals become more aware of their values through an increased awareness of everyday thoughts, emotions and sensations, existential psychology (through the implementation of philosophy activities for children) was added so that children could directly and actively reflect on their personal values, facilitating the process of identifying them. Integrating mindfulness and existential psychology could thus provide children with a more effective intervention to foster self-determination, as defined by SDT. More concretely, by practicing mindfulness exercises, a child could start noticing that their throat gets tight when they are in the presence of a classmate whom they call a friend, or that they feel warm and tingly when they think about their pet. This could help them become more aware of what is important to them (e.g., animals, being with people they love). In turn, this knowledge could foster a much deeper reflection and integration for them when they engage in the philosophy activities.

## Philosophy for Children (P4C)

The roots of existential psychology can be traced back to philosophy, which has aimed for centuries to provide answers to humans' need to make sense of the world (42). Similarly, children, as much as adults, strive to make sense of the world surrounding them (45). Philosophy for children (P4C), centered on the practice of dialogue, aims to develop children's capacity to think by and for themselves and to become more self-determined, providing some answers to questions children may ask themselves (46–48). As such, P4C aims primarily to provide an educational context in which children are asked to think by and for themselves about topics that are of interest to them, fostering greater intellectual and emotional autonomy, and consequently, higher self-determination.

Such aims converge toward the prospective and utopic functions of philosophical dialogue as a practice, broadly defined as the capacity for students to understand themselves as subjects acting upon the world. Some educational researchers link this to the concept of agency or to the idea that attitudes, methods, and cognitive processes subsumed by the philosophical dialogues contribute to pupils' recognition of the power of individual and collective agency to act upon the present and to mold the future. They are likely to lead pupils to see themselves as the subjects of a complex history, influenced, at least partly, by their thoughts and actions through the practice of dialogue (49, 50).

P4C is becoming increasingly popular in school settings, namely to foster greater well-being in youth (47). Its practice aims to foster greater awareness of thoughts and emotions, and, as such, shares common goals with mindfulness (51, 52). During P4C activities, students are presented with an existential or philosophical theme (while reading a story, viewing a short video, or choosing specific themes amongst themselves) (53). Following the initial philosophical primer, students are invited to identify, as a group, topics they wish to discuss (53–56). In this philosophical process, pupils are expected to learn to question, conceptualize and problematize natural or human phenomena, as well as to deconstruct, rationally weigh, take position on and tolerantly discuss thoughts related to existential, ethical, esthetical, epistemological, etc., controversies.

There is a paucity of evidence-based, experimental results on the impact of P4C activities on mental health in children (52). Available correlational and quasi-experimental results indicate a positive association between P4C, cognitive and academic skills (57, 58), self-esteem, and the ability to identify one's own emotions and thoughts in elementary school children (59). Upon researching the literature on this topic, only two quasi-experimental studies documenting the association between P4C techniques and children's well-being were found. One study among children displaying various emotional and behavioral disorders (including, but not restricted to, autism spectrum disorders, and communication disorders) in a special education curriculum reported benefits in self-regulatory (namely turn-taking and patience) and overall participation in class in these participants (60). Further work by the same group of authors showed similar results on self-regulation and rule adherence among children with severe behavioral disorders receiving special education in secure settings (61). While these correlational results provide preliminary evidence for the use of P4C activities in classrooms, to our knowledge, no experimental study has been published on the impact of P4C on mental health and well-being in children. Furthermore, there is no available, published evidence of the influence of P4C on self-determination and basic psychological needs satisfaction. One goal of this study was thus to implement an experimental design to document the impact of P4C in pre-kindergarten children using a randomized controlled trial and to evaluate if P4C had an impact on self-determination in children.

### Present Study

Based on the complementarity between existential psychology and mindfulness interventions for youth, we designed an intervention combining a MBI and P4C activities for pre-kindergarten children. The main objective was thus to conduct a pilot feasibility study exploring the effectiveness of an intervention combining mindfulness-based and P4C activities. We evaluated the impact of this combined intervention on positive (i.e., social skills and adaptability) and negative (i.e., internalized symptoms: depression, anxiety, inattention; and hyperactivity) indicators of well-being using an experimental cluster design with a wait-list control condition.

### Hypotheses

Based on previous findings from the literature on MBIs in youth and preliminary literature on the impact of P4C, we hypothesized that the combined intervention would have a beneficial impact on participants' psychological health and well-being. Specifically, we hypothesized children from the experimental group would experience increases in positive indicators of well-being (i.e., better social skills and adaptability) from pre-to-post intervention. These increases would be greater than those observed in children from the control group. We also hypothesized that children from the experimental group would experience decreases in negative indicators of psychological health (i.e., internalized symptoms) from pre-to-post intervention, and that these decreases would be greater than those observed in children from the control group.

## Methods

Two classrooms of pre-kindergarten children (*N* = 38, mean age = 4.6 years-old) from a private elementary school in the Eastern Townships area (Quebec, Canada) took part in this study and were randomly allocated to an experimental (*n* = 19 students) or a wait-list control condition (*n* = 19 students). Descriptive statistics can be found in Table 1. A randomized cluster trial with a wait-list control group was implemented to evaluate the impact of the combined MBI and P4C intervention on positive and negative indicators of well-being. Teachers completed measures at pre-intervention and immediately post-intervention. There was no attrition in this study. After the intervention was completed with the experimental group, wait-list controls were offered the intervention. Informed consent was obtained from all parents of students taking part in this study, as well as from both teachers participating. All parents gave consent for their child to participate in the project.

**Table 1:** Descriptive statistics and sample distribution.

	***N***	**Mean age**	**Condition**
Experimental group	19	4.6	MBI and P4C
Control group	19	4.7	Wait-list
Total sample	38	4.68	
Girls	23		
Boys	15		

### Procedure

A 5-weeks intervention was implemented as part of this project. Specifically, each weekly session comprises a mindfulness opening activity, followed by a P4C activity. Each session lasted ~1 h. Mindfulness activities comprised in this intervention were: (1) an introductory activity on mindfulness meditation and related psychoeducational content about thoughts, physical sensations and emotions; (2) mindful eating; (3) mindful pausing; (4) mindful listening; and (5) mindfulness and gratitude. The mindfulness activities were adapted from the *Mission Meditation* manual, an evidence-based intervention adapted and tailored to fit elementary school children's developmental needs and attention span (4). P4C activities comprised in this intervention were based on themes of: (1) happiness; (2) normal vs. not normal; (3) making mistakes; (4) sadness and anger; and (5) separation and death. These themes were selected as they were most closely related to existential psychology and were thought to foster self-determination more directly in children. Students were presented with short stories, simple questions (often accompanied by an image or poster to illustrate the question), short comic strips and video clips. The intervention was led by two undergraduate students in psychology with extensive training in MBIs and P4C. Both were supervised on a weekly basis by the research team throughout the course of this project.

### Measure

Given the age of the participants in this study, teachers were asked to complete the pre-intervention and immediately post-intervention questionnaires. Specifically, teachers were asked to report on children's anxiety (three items, e.g., Worries about things that cannot be changed), depression (three items, e.g., Is sad), inattention (three items, e.g., Has a short attention span), hyperactivity (three items, e.g., Has trouble staying seated), social skills (three items, e.g., Encourages others to do their best), and adaptability (three items; Adjusts well to changes in routine; Is easily calmed when angry; Seems to take setbacks in stride) using the respective subscales of the *Behavior Assessment Scale for Children* (BASC III) (62). The teacher-report form was used to evaluate the presence of internalized and externalized symptoms in children. Teachers were asked to rate their agreement on a 4-point Likert-type scale (1–never to 4–always) and subscale scores were summed. For the purposes of the present study, depression, anxiety and inattention were grouped to represent overall changes in internalized symptoms. Internal consistency was acceptable to good for the majority of subscales in this sample (α _adaptability_ = 0.78; α _socialskills_ = 0.69; α _hyperactivity_ = 0.80; α _internalizedsymptoms_ = 0.81).

### Data Analysis

Hypotheses were tested using ANCOVAs, as they have been recommended to increase statistical power in randomized controlled trials (63). In this study, ANCOVAs allowed the comparison of post-intervention scores across groups, while controlling for pre-intervention scores. *Post-hoc* analyses were conducted using paired t-tests, to evaluate pre-to-post differences in scores within each group and examine changes over time. Effect sizes were also computed in order to assess the magnitude of the observed effects.

## Results

Preliminary analyses using independent *t*-tests first showed that the two groups differed at pre-intervention on measures of social skills [*t*_(37)_ = −3.60, *p* = 0.001] and adaptability [*t*_(37)_ = −4.49, *p* < 0.001]. In other words, children from the experimental group had higher levels of social skills and adaptability than the children from the control group. Descriptive results can be found below and in Tables 2, 3. Data assumptions for normality (64), homogeneity of variance (65), and sphericity were all met in this sample.

**Table 2:** Means and standard deviations for negative and positive indicators of well-being (scores on the BASC-III subscales).

	**Control group**		**Experimental group**	
**Dependent variable**	**Pre-test** **(SD)**	**Post-test** **(SD)**	**Pre-test** **(SD)**	**Post-test** **(SD)**
**Negative indicators**				
Internalized symptoms	9.29 (3.84)	7.88 (4.74)	9.61 (2.03)	8.55 (2.61)
Hyperactivity	3.37 (1.77)	2.26 (2.08)	4.08 (1.78)	3.55 (1.64)
**Positive indicators**				
Social skills	2.11 (2.35)	3.16 (2.17)	4.16 (0.94)	3.66 (1.07)
Adaptability	3.21 (1.47)	3.74 (1.33)	4.89 (0.81)	5.00 (1.27)

**Table 3:** Results of ANCOVA for internalized symptoms, hyperactivity, social skills and adaptability.

**Variable**	**df**	**F**	***p***	**Partial η^2^**
Internalized symptoms	1	2.08	0.15	0.05
Hyperactivity	1	2.81	0.10	0.07
Social skills	1	1.07	0.32	0.02
Adaptability	1	0.49	0.48	0.01

### Main Analyses

#### Negative Indicators of Mental Health

ANCOVAs did not reveal a significant effect of condition on internalized symptoms [*F*_(1, 33)_ = 2.08, *p* = 0.15, partial η^2^ = 0.05], controlling for baseline levels. Sensitivity analyses were conducted using paired *t*-tests. These analyses indicated that for the whole sample, internalized symptom scores were statistically significantly lower at post-intervention [*t*_(35)_ = 2.45, *p* = 0.01; 95% CI = 0.23, 2.53], when compared to pre-intervention scores. Hence, participants from both groups displayed lower internalized symptom scores from pre-to-post intervention, descriptive statistics showing a similar decrease in scores for participants from both groups.

We found no impact of group on levels of hyperactivity [*F*_(1, 37)_ = 2.81, *p* = 0.10, partial η^2^ = 0.07; please refer to Table 3). Sensitivity analyses were conducted using paired *t*-tests. These analyses indicated that for both the experimental and control groups, hyperactivity scores were statistically significantly lower at post-intervention [*t*_(37)_ = 3.24, *p* = 0.002; 95% CI = 0.30, 1.32], when compared to pre-intervention scores. Hence, participants from both groups displayed lower hyperactivity scores from pre-to-post intervention, descriptive statistics showing a similar decrease in scores for participants from both groups.

#### Positive Indicators of Mental Health

We found no impact of group on levels of social skills [*F*_(1, 37)_ = 1.01, *p* = 0.32, partial η^2^ = 0.02] and adaptability [*F*_(1, 37)_ = 0.49, *p* = 0.48, partial η^2^ = 0.01; please refer to Table 3].

### Change Over Time

#### Negative Indicators of Mental Health

To document change over time, we conducted paired *t*-tests to examine within-group changes in pre-to-post intervention scores. For internalized symptoms, paired *t*-tests revealed a statistically significant change within participants in the experimental group [*t*_(18)_ = 2.19, *p* = 0.04] and the wait-list control group [*t*_(16)_ = 2.29, *p* = 0.03]. Participants from both groups showed a decrease in internalized symptoms from pre-intervention (M_preexperimental_ = 9.61; M_precontrol_ = 9.29) to post-intervention (M_postexperimental_ = 8.55; M_postcontrol_ = 7.88). Similarly, for hyperactivity, paired t-tests revealed statistically significant change within participants in the in the experimental group [*t*_(18)_ = 2.45, *p* = 0.02] and the wait-list control group [*t*_(18)_ = 2.44, *p* = 0.02]. Participants from both groups showed a decrease in internalized symptoms from pre-intervention (M_preexperimental_ = 4.08; M_precontrol_ = 3.37) to post-intervention (M_postexperimental_ = 3.55; M_postcontrol_ = 2.26).

#### Positive Indicators of Mental Health

Paired t-tests did not reveal statistically significant pre-to-post changes for adaptability (experimental: *t*_(18)_ = −0.36, *p* = 0.71; control: *t*_(18)_= −1.88, *p* = 0.70) for participants in the experimental and wait-list control groups. For social skills, paired t-tests did not reveal statistically significant pre-to-post changes in participants from the experimental group [*t*_(18)_ = 1.52, *p* = 0.14). In contrast, paired t-tests were significant for participants from the control group [*t*_(18)_ = −2.4, *p* = 0.02]. Hence, participants in wait-list control group showed an increase in social skills scores from pre-intervention (M_pre_ = 2.11) to post-intervention (M_post_ = 4.16), whereas scores remained similar among participants in the experimental group (M_pre_ = 3.16; M_post_ = 3.66).

## Discussion

The goal of the present study was to evaluate the impact and feasibility of a combined MBI and P4C intervention on negative and positive indicators of mental health. Overall, results from this pilot feasibility study show that the combined MBI and P4C intervention was not more useful in decreasing internalized symptoms, hyperactivity symptoms, social skills and adaptability as the passage of time, as can be seen by similar improvements in scores of the control group participants and small effect sizes. These preliminary results suggest that mindfulness and P4C may not be more effective as an intervention to foster short-term resiliency, well-being and mental health in preschool children as the passage of time.

As detailed in the introduction, the initial goal of this pilot feasibility study was to explore the impact of a combined mindfulness and P4C intervention on positive and negative indicators of mental health in pre-kindergarten children, with the overarching goal of developing an adaptation of the existential psychology framework for youth. The present findings are surprising given that previous research had shown a positive impact of both interventions with elementary school children (5, 57, 58, 60).

Given the paucity of evidence-based research pertaining to the usefulness of P4C on mental health, results from this pilot are difficult to explain in relation to the previous literature. Indeed, upon researching the literature on this topic, one quasi-experimental study documenting the impact of P4C on mental health in children (aged between 9 and 12 years-old) with severe behavioral disorders was found and benefits with regards to self-regulation were reported (60). Work by the same group of authors also show improvements in self-regulation and rule adherence following P4C in teenagers with severe psychiatric disorders in juvenile prison settings (61). Authors argue that, although encouraging dialogue and argumentation in juvenile prisons may be perceived as counterintuitive, the use of P4C for these teenagers may be helpful in fostering a sense of control and self-determination in an environment in which typically there is none. As such, P4C may show promise in fostering greater autonomy and self-determination in this specific population. Self-determination theory would also allow for the empirical study of themes pertaining to existential psychology with youth.

Caution is warranted in drawing conclusions between the studies mentioned above, implemented with older elementary school students, and the present one, implemented with pre-kindergartners, because of the important difference in ages and levels of cognitive, social and emotional development. Nonetheless, it is worth mentioning that P4C activities chosen for this study were initially developed for pre-kindergartners and adapted accordingly to their cognitive, social and emotional levels of reasoning. Furthermore, past research in philosophy has also been published on this practice for pre-kindergartners and kindergartners alike (51, 66), suggesting P4C is adequate and may be beneficial with younger children as well.

An appreciable amount of research describing the positive impact of MBIs on positive and negative indicators of mental health in clinical and non-clinical samples of elementary school children has been published previously (5, 12, 67). Although research with pre-kindergarteners remains scarce, previous work by Flook et al. (68) has described benefits of mindfulness training with regards to social skills, social-emotional development and overall academic achievement for this population. On the other hand, recent work by Thierry et al. (69), failed to find statistically significant impacts of mindfulness training on prosocial behaviors in prekindergartners, although authors did report benefits with regards to executive functioning (working memory, inhibition and flexibility). Overall, these results seem to indicate that MBIs are a suitable intervention for preschoolers, although the present study does not show the same results.

We did not find similar results in the present study and the reason for this remains unclear, although certain explanations warrant attention. Some studies on mindfulness have documented ceiling effects in non-clinical populations in school settings (70), which may partly explain the present findings. Speaking to the low-level baseline of psychopathology in this sample, the available margins for change and for overall improvement from pre-to-post intervention were lower, which could explain the non- statistically significant results and small effect sizes that were obtained. Indeed, a recent meta-analysis on the impact of mindfulness-based interventions in youth showed much stronger effect sizes among clinical populations (0.50 vs. 0.20 for non-clinical populations), as there is more “room to improve” among these children (67). Nonetheless, it is important to note that participants from both the control and experimental groups showed improvements from pre-to-post intervention.

This being said, a recent report on Canadians' mental health has recommended that more research be devoted to developing evidence-based interventions that can be universally implemented in classroom as prevention strategies to foster better mental health and to prevent the development of psychological disorders in elementary school students (71). Cost-benefit analyses of the best mental health prevention programs for youth in the United States have also shown that these programs yield a minimum of 8$ for each dollar invested in mental health prevention. Thus, investing in developing prevention programs is a viable and effective solution to decrease future potential mental health issues and psychological disorders in school-based settings (72).

Furthermore, some methodological issues may have influenced the results. Noticing internalized symptoms (and subsequent changes in these symptoms) in students is known to be a difficult task for teachers, given that they are not as disruptive as externalized symptoms and do not tend to impact the overall classroom climate as much (17). Teachers were also not blind to both conditions in this study, which could have biased their way of reporting pre and post intervention scores for their students. In order to correct for these important issues, adding parent-reported data in future studies of this sort could help in providing a clearer picture of the overall evolution of internalized symptoms in preschool children participants. It could also be interesting for future research to include behavioral measures of constructs such as altruism (e.g., to give children a number of stickers and ask them how many they would keep and share with others), in order to reduce shared method variance.

Another reason that could explain our results is the duration of our intervention. Implementing a longer intervention, such as the 10-weeks intervention detailed in the abovementioned studies, could also perhaps yield different results, as the “dosage” would be stronger. Lasting only 5 weeks, this combined intervention was considered as brief and therefore may not have been “strong” enough to provoke changes among the children. Finally, it is possible that philosophical activities are a source of stress in very young children, which may not have developed the cognitive ability to reflect on existential questions. It is also possible that these manifestations of stress are short-lived and are worth it for future gain. Anecdotal evidence provided by participants and their teachers alike tend to support the latter. Indeed, teachers did not report an increase in distress in their students, and students reported liking to participate in the P4C activities. They were eager for the next visit from session leaders and asked frequently their teachers when they would be back in class.

From a feasibility standpoint, results from this study and anecdotal evidence collected from teachers taking part in this project and from session leaders show the intervention was positively received and enjoyed by students and teachers alike. Themes were understood by the participants, who actively participated in the MBI and P4C activities. Visual and video content was preferred to stories and comic strips, as children could easily and rapidly understand the existential themes that were presented to them. When stories were used, session leaders reported they often had to reword the content of the story and remind students of the theme during the discussion. In such instances, participants did not share amongst themselves as much, which made for less lively conversations. Perhaps the use of philosophically-oriented stories may be more adequate for older elementary school children. Overall, however, the project was well received by children and the school and thus, shows good acceptability and feasibility. This underlines the need for more research, as anecdotal evidence and empirical evidence brought forward by the data, such as the small effect sizes, are contradictory in this study.

### Strengths and Limitations

This study counts multiple strengths. First, the experimental design implemented in this project represents a notable strength. Indeed, both classrooms were similar at pre-intervention, with the exception of the level of social skills and adaptability, and were randomly assigned to each condition. With regards to social skills and adaptability, given pre-intervention scores from students in the experimental group were statistically and significantly higher than those of students in the control group, it is possible the control group simply “caught up” with the other group over time. However, with regards to social skills and adaptability, although children in the experimental group were substantially higher than those in the control group prior to the intervention, at post intervention, when looking at the confidence intervals, both groups overlapped, and thus were not really different from each other. Second, the absence of attrition in this study further represents a major strength, as it ensures pre-to-post differences in scores and changes in time are not due to changes in the sample. Finally, this study provides preliminary data on the impact of P4C sessions (combined with the MBI) from the rigorous standpoint of an experimental, randomized cluster trial. Given almost-to-none evidence-based data is available on the impact of P4C in preschool and elementary school children in psychology, this study contributes to strengthening the knowledge on this increasingly implemented practice in classrooms across North America. Finally, this study was innovative in its aim to document the adaptation of the existential psychology framework for children, although it could not provide preliminary evidence showing its promise in fostering greater mental health and well-being. Future work will be needed to establish if such benefits are observed in school settings.

Despite these strengths, the small sample size represents a noticeable limitation of this project. As it remains possible that both classrooms may have been different, a larger randomized cluster trial would have provided more robust and generalizable results, and should thus be planned as future steps in this line of research. This would also provide higher statistical power. Implementing a longitudinal trial would also strengthen future studies of this sort, as results from the present study only speaks to immediate, post-intervention effects of the intervention on children's mental health. Past mindfulness research in youth has shown patterns where benefits become apparent only after a 3 or 6-months follow-up (35). Similar patterns could emerge in our participants, although this remains a hypothesis at the moment. Administering a more important number of items in future studies may also be helpful in providing a clearer, more complete portrait of the situation. Indeed, as only a few selected items of each subscale were administered in the current project, this reduced the sensitivity of our measures to changes in our participants. Perhaps new statistically significant pre-to-post changes would be detected with a larger number of administered items. We note, however, that with this sample size and this number of items, we were still able to detect changes in internalized symptoms, hyperactivity and social skills. Furthermore, all questionnaires in one group were filled out by one teacher, which may have further biased our results. Finally, given improvements that were observed in participants from both conditions, it remains a strong possibility that the documented effects were due to the simple passage of time.

### Suggestions for Further Research

Adding a longitudinal component to future studies of the sort would allow for a more detailed portrait of the impact of such a combined intervention on mental health in youth. Furthermore, using age as a moderating variable in larger sample size studies would also allow us to better grasp if P4C is most useful with younger or older elementary school children. Based on previous preliminary research in P4C with youth from clinical populations and our own justification of the relevance of combining a MBI and P4C, measuring self-determination in future studies is also recommended, as, theoretically, a combined mindfulness and P4C intervention should help increase self-determination in children. Furthermore, as P4C activities are often led by teachers or completed in the presence of teachers, future studies could incorporate observational methods to control for teacher autonomy supportive behaviors (73), as these behaviors have been linked to higher reported levels of basic psychological needs satisfaction in children and overall greater self-determination (74, 75).

Similarly, future studies of this sort could incorporate observational methods to evaluate changes in altruism, as similar methods have been successfully previously employed with preschoolers in the mindfulness literature (68). Implementing a multi-method approach by combining observational and teacher-reported measures would increase the scope of variables being evaluated and strengthen the study design. Including parents and caregivers reported data would also be useful in gaining better insight of the impact this combined intervention has in pre-kindergarten children.

Finally, a further step would also be to compare the effectiveness of the combined intervention to another intervention aimed at fostering greater mental health and self-determination in children, within an experimental design with an active control group. Meanwhile, publishing any evidence-based data on the impact of P4C would also help in providing a better sense of its usefulness (or lack thereof) on psychological health and well-being in preschoolers youth.

## Conclusion

Overall, results from this pilot feasibility study showed that the MBI and P4C intervention was not more useful in decreasing anxiety and hyperactivity symptoms as the passage of time, as can be seen by similar or greater improvements in scores of the control group participants. These preliminary results suggest that mindfulness and philosophy for children may not be the most effective intervention to foster short-term resiliency, well-being and better mental health in children. Further research using longer time frames and comparing the effectiveness of this combined intervention to other types of school-based interventions with similar aims (such as, for example, P4C or MBI alone) is warranted, to evaluate if mindfulness and P4C interventions have an added value compared to other types of interventions implemented in school settings.

## Data Availability Statement

The datasets generated for this study are available on request to the corresponding author.

## Ethics Statement

The studies involving human participants were reviewed and approved by Bishop's University Research Ethics Board. Written informed consent to participate in this study was provided by the participants' legal guardian/next of kin and by the teachers taking part in this study.

## Author Contributions

CM-H and DL conceptualized and coordinated the study, adapted the intervention and trained the session leaders involved in this study, performed data analysis, and drafted the manuscript. GM and GT contributed extensively to data interpretation and revision of the manuscript. CD helped in data collection and coordination of the study, while contributing to revision of the manuscript. M-AE and MG contributed to the design of the study and revision of the manuscript. All authors contributed to the article and approved the submitted version.

## Conflict of Interest

CM-H has released a manual on the mindfulness-based intervention described and used in this study (Midi Trente Publishers). The remaining authors declare that the research was conducted in the absence of any commercial or financial relationships that could be construed as a potential conflict of interest.
